# Disparities in Enrollment in Medicare Advantage Plans With Dental Benefits

**DOI:** 10.1001/jamanetworkopen.2023.56095

**Published:** 2024-02-14

**Authors:** Darien J. Weatherspoon, Susan Hutfless, Richard J. Manski, John F. Moeller

**Affiliations:** 1 Department of Dental Public Health, University of Maryland School of Dentistry, Baltimore; 2 Custom Data Shop, Conshohocken, Pennsylvania

## Abstract

This cross-sectional study evaluates the association of Medicare beneficiaries’ sociodemographic characteristics with having Medicare Advantage plans that cover oral health services.

## Introduction

Traditional fee-for-service Medicare rarely covers oral health services.^1^ Medicare beneficiaries in racial and ethnic minority groups and with lower socioeconomic status (SES) are less likely to have recent dental visits than their counterparts.^2^ Unlike traditional Medicare, most Medicare Advantage (MA) plans offer access to oral health services,^2^ and enrollment in MA has been highest among historically underserved adults 65 years or older.^3^ In this study, we aimed to assess the prevalence and likelihood of having MA plans offering dental coverage among beneficiaries by sociodemographic factors.

## Methods

A cross-sectional design was used to analyze dental coverage across the MA population. Beneficiaries with 12 months of Part C (MA) coverage were included. The University of Maryland Baltimore Institutional Review Board deemed the study exempt from review and informed consent because it was non–human participant research. We followed the STROBE reporting guideline.

Three categories of dental coverage were constructed from dental benefits data in the Centers for Medicare and Medicaid Services 2019 Plan Benefit Package (PBP): no coverage, preventive only, and comprehensive. Dental coverage was not distinguished as optional or mandatory. The 2019 PBP quarterly data were linked to 2019 Master Beneficiary Summary File to determine prevalence of each level of dental coverage across sociodemographic characteristics of Medicare beneficiaries. Race and ethnicity were based on self-reported data from the Social Security Administration and subsequent linkage to Medicare enrollment data.

A multivariable logistic regression model was used to estimate odds of having MA plans offering any dental coverage (preventive only or comprehensive) across demographic groups. Sensitivity analyses were conducted to ascertain whether these odds changed when having a plan with mandatory dental coverage was assessed as the outcome. Data analysis was performed between September and December 2023 using SAS 9.4 (SAS Institute).

## Results

There were 21 219 195 Medicare beneficiaries (12 029 307 females [56.7%], 9 189 888 males [43.3%]; 18 590 043 aged ≥65 years [87.6%]) with 12 months of MA coverage in 2019 (Table 1). Overall, 32.7% of beneficiaries had an MA plan with no coverage, 14.0% with preventive only, and 53.3% with comprehensive coverage. Compared with beneficiaries in other demographic categories, beneficiaries with a higher prevalence of no dental coverage were 75 years or older (36.8%), White individuals (34.9%), not dual-eligible (37.3%), and qualified for Medicare because of age (36.7%). Dental coverage prevalence did not vary appreciably by sex.

**Table 1.** Demographic Characteristics and Dental Coverage of 2019 Medicare Advantage Beneficiaries

Characteristics	Dental coverage, No. (%)^a^			Total No.
	No	Preventive only	Comprehensive	
All beneficiaries	6 936 697 (32.7)	2 961 938 (14.0)	11 320 560 (53.4)	21 219 195
Age, y				
≤64	386 728 (14.7)	330 146 (12.6)	1 912 278 (72.7)	2 629 152
65-74	3 395 700 (33.9)	1 445 105 (14.4)	5 171 630 (51.7)	10 012 435
≥75	3 154 269 (36.8)	1 186 687 (13.8)	4 236 652 (49.4)	8 577 608
Sex				
Female	3 965 551 (33.0)	1 660 546 (13.8)	6 403 210 (53.2)	12 029 307
Male	2 971 146 (32.3)	1 301 392 (14.2)	4 917 350 (53.5)	9 189 888
Race and ethnicity^b^				
American Indian or Alaska Native	11 458 (24.7)	6342 (13.7)	28 572 (61.6)	46 372
Asian	161 189 (26.7)	64 914 (10.7)	378 201 (62.6)	604 304
Black	681 909 (25.3)	286 174 (10.6)	1 724 229 (64.0)	2 692 312
Hispanic	143 325 (16.6)	60 448 (7.0)	660 199 (76.4)	863 972
White	5 640 014 (34.9)	2 436 461 (15.1)	8 081 608 (50.0)	16 158 083
Unknown	124 840 (34.6)	54 813 (15.2)	181 290 (50.2)	36 0943
Other^c^	173 962 (35.3)	52 786 (10.7)	266 461 (54.0)	493 209
Dual eligible				
No	6 330 324 (37.3)	2 596 357 (15.3)	8 030 048 (47.4)	16 956 729
Yes	606 373 (14.2)	365 581 (8.6)	3 290 512 (77.2)	4 262 466
Original reason for Medicare qualification				
Age	5 954 223 (36.7)	2 317 619 (14.3)	7 938 349 (49.0)	16 210 191
Disability	972 334 (19.6)	641 004 (12.9)	3 359 591 (67.6)	4 972 929
ESKD alone	5181 (31.6)	1267 (7.7)	9970 (60.7)	16 418
ESKD and disability	4959 (25.2)	2048 (10.4)	12 650 (64.4)	19 657
ESKD alone or ESKD and disability	10 140 (28.1)	3315 (9.2)	22 620 (62.7)	36 075

Abbreviation: ESKD, end-stage kidney disease.

^a^ Percentages may not add to exactly 100% due to rounding.

^b^ Race and ethnicity are based on self-reported data from the Social Security Administration and include those categories listed in the Master Beneficiary Summary File. Racial groups exclude beneficiaries who are of Hispanic ethnicity. Beneficiaries of Hispanic ethnicity are categorized as Hispanic. Beneficiaries for whom race and ethnicity data were missing were included in the unknown category.

^c^ Other includes race and ethnicity outside of the categories specified in the Master Beneficiary Summary File.

Odds of having MA plans offering any dental coverage were different across demographic groups (Table 2). Beneficiaries 64 years or younger had higher odds of having dental coverage than those aged 65 to 74 years (adjusted odds ratio [AOR], 1.57; 95% CI, 1.56-1.58). Racial and ethnic minority beneficiaries generally had higher odds of having dental coverage than White beneficiaries, with Hispanic individuals displaying the highest AOR (1.71; 95% CI, 1.70-1.72). Dual-eligible beneficiaries had higher odds of having dental coverage compared with non-dual-eligible beneficiaries (AOR, 2.77; 95% CI, 2.76-2.77). Individuals qualifying for Medicare due to disability had higher odds than beneficiaries qualifying because of age (AOR, 1.46; 95% CI, 1.46-1.47). In the regression model with mandatory coverage as the outcome, the overall findings were similar; however, the magnitude of many estimates were attenuated.

**Table 2.** Any Dental Coverage in 2019 Medicare Advantage (MA) Plan for Select Demographic Characteristics^a^

Characteristic	Any dental coverage in MA plan, AOR (95% CI)^b^
Age, y	
≤64	1.57 (1.56-1.58)
65-74	1 [Reference]
≥75	0.92 (0.91-0.92)
Sex	
Female	0.95 (0.94-0.95)
Male	1 [Reference]
Race and ethnicity^c^	
American Indian or Alaska Native	1.21 (1.18-1.23)
Asian	1.23 (1.22-1.24)
Black	1.18 (1.17-1.18)
Hispanic	1.71 (1.70-1.72)
White	1 [Reference]
Other^d^	0.97 (0.97-0.98)
Dual-eligible status	
No	1 [Reference]
Yes	2.77 (2.76-2.77)
Original reason for Medicare qualification	
Age	1 [Reference]
Disability	1.46 (1.46-1.47)
ESKD alone	0.71 (0.68-0.73)
ESKD and disability	0.92 (0.89-0.95)

Abbreviations: AOR, adjusted odds ratio; ESKD, end-stage kidney disease.

^a^ Any dental coverage was defined as having an MA plan offering either preventive only or comprehensive dental coverage.

^b^ Logistic regression model included age, sex, race and ethnicity, dual-eligible status, and original reason for Medicare qualification.

^c^ Race and ethnicity are based on self-reported data from the Social Security Administration and include categories listed in the Master Beneficiary Summary File. Racial groups exclude beneficiaries who are of Hispanic ethnicity. Beneficiaries of Hispanic ethnicity are categorized as Hispanic.

^d^ Other includes races and ethnicities outside of the categories specified in the Master Beneficiary Summary File.

## Discussion

The results suggest that almost one-third of 2019 Medicare beneficiaries had MA plans without dental coverage. Consistent with a previous study using 2016 data,^4^ this study found that beneficiaries in racial and ethnic minority groups and with lower SES were more likely to have plans offering dental coverage than their counterparts; associations were also observed between other demographic factors and dental coverage.

A study limitation is that we did not control for geography, which can affect who has access to MA plans with dental coverage. Because recent MA enrollment has been highest among traditionally underserved groups,^4^ and many of these individuals have plans offering dental coverage, MA could play a role in increased access to dental care and subsequent reduction in oral health disparities among Medicare beneficiaries.^5,6^

## Notes

Supplement.
Data Sharing Statement
