# Supplementary material for: Disparities in Enrollment in Medicare Advantage Plans With Dental Benefits
Source: JAMA Netw Open. 2024 Feb 14;7(2):e2356095. doi: 10.1001/jamanetworkopen.2023.56095 (PMC10867677; doi:10.1001/jamanetworkopen.2023.56095)
Supplement: Supplement. — Data Sharing Statement [file jamanetwopen-e2356095-s001.pdf]

Data Sharing Statement

Weatherspoon. Disparities in Enrollment in Medicare Advantage Plans With Dental Benefits. *JAMA Netw Open*. Published February 14, 2024. doi:10.1001/jamanetworkopen.2023.56095

Data
Data available: No

Additional Information
Explanation for why data not available: The data cannot be provided because access to the data requires a data usage agreement with the Centers for Medicare & Medicaid Services
